# Differential responses of sugar, organic acids and anthocyanins to source-sink modulation in Cabernet Sauvignon and Sangiovese grapevines

**DOI:** 10.3389/fpls.2015.00382

**Published:** 2015-05-29

**Authors:** Natalia Bobeica, Stefano Poni, Ghislaine Hilbert, Christel Renaud, Eric Gomès, Serge Delrot, Zhanwu Dai

**Affiliations:** ^1^INRA, ISVV, EGFV, UMR 1287, University of BordeauxVillenave d’Ornon, France; ^2^Fruit Culture and Viticulture Section, Department of Sustainable Crop Production, Università Cattolica del Sacro CuorePiacenza, Italy

**Keywords:** *Vitis vinifera* L, wine alcohol content, wine color, climate change, leaf-to-fruit ratio, berry composition

## Abstract

Grape berry composition mainly consists of primary and secondary metabolites. Both are sensitive to environment and viticultural management. As a consequence, climate change can affect berry composition and modify wine quality and typicity. Leaf removal techniques can impact berry composition by modulating the source-to-sink balance and, in turn, may mitigate some undesired effects due to climate change. The present study investigated the balance between technological maturity parameters such as sugars and organic acids, and phenolic maturity parameters such as anthocyanins in response to source-sink modulation. Sugar, organic acid, and anthocyanin profiles were compared under two contrasting carbon supply levels in berries of cv. Cabernet Sauvignon and Sangiovese collected at 9 and 14 developmental stages respectively. In addition, whole-canopy net carbon exchange rate was monitored for Sangiovese vines and a mathematic model was used to calculate the balance between carbon fixation and berry sugar accumulation. Carbon limitation affected neither berry size nor the concentration of organic acids at harvest. However, it significantly reduced the accumulation of sugars and total anthocyanins in both cultivars. Most interestingly, carbon limitation decreased total anthocyanin concentration by 84.3% as compared to the non source-limited control, whereas it decreased sugar concentration only by 27.1%. This suggests that carbon limitation led to a strong imbalance between sugars and anthocyanins. Moreover, carbon limitation affected anthocyanin profiles in a cultivar dependent manner. Mathematical analysis of carbon-balance indicated that berries used a higher proportion of fixed carbon for sugar accumulation under carbon limitation (76.9%) than under carbon sufficiency (48%). Thus, under carbon limitation, the grape berry can manage the metabolic fate of carbon in such a way that sugar accumulation is maintained at the expense of secondary metabolites.

## Introduction

Grapevine is an important perennial crop cultivated in many countries (7519 mha in 2013; OIV, 2014). Its fruits are used predominantly for wine making, yet also for juice, raisins and fresh consumption. Grape berry composition, which is important for the grape growers and the wine industry, is mainly determined by sugars, organic acids, and various secondary metabolites (e.g., tannins, flavonols, anthocyanins, aroma precursors, and volatile compounds; Conde et al., 2007). The accumulation of these components along berry development and ripening depends on the genotype and on the environment (Jackson and Lombard, 1993).

Climate change already affects the physiology of the grapevine (Schultz, 2000), causing increased sugar concentration and, consequently, higher alcohol content in wines (Duchêne and Schneider, 2005; Bock et al., 2013), reduced organic acids and anthocyanins (Barnuud et al., 2013, 2014), and modified aroma profiles (Keller, 2010a). In the long term, the sustainability of wine production in several viticultural regions may be threatened by climate change (Schultz and Jones, 2010; Hannah et al., 2013). To face such challenges, the mechanisms controlling the accumulation of quality-related metabolites in grapes must be better understood. This will allow promoting innovative viticultural practices resulting in easier adaptation of wine production to climate change (van Leeuwen et al., 2013).

Among the different viticultural practices affecting berry composition (Keller, 2010a; Dai et al., 2011; Kuhn et al., 2014), source-sink modulation by summer pruning (i.e., leaf removal or shoot and cluster thinning) is an important tool that may control the relationship between yield and quality, and adjust the complex chemical composition of grape berry (Kliewer and Dokoozlian, 2005). For example, the berry sugar concentration is often positively correlated with leaf area-to-yield ratio when the ratio is below a threshold value of about 1 m^2^/Kg of fruit mass (Kliewer and Dokoozlian, 2005; Duchêne et al., 2012). Above this value, the sugar concentration usually reaches a plateau and becomes less responsive to source-sink modulation (Kliewer and Dokoozlian, 2005). The responses of organic acids to source-sink modulation have been less thoroughly studied, and contradictory reports showed that a lower leaf area-to-yield ratio caused either an increase (Wolpert et al., 1983; Ollat and Gaudillere, 1998; Wu et al., 2013), decline (Bravdo et al., 1985), or lack of response (Reynolds et al., 1994; Parker et al., 2015) of organic acids compared with a high leaf to yield ratio.

In addition to primary metabolites, secondary metabolites (e.g., tannins, flavonols, anthocyanins, aroma precursors, and volatile compounds) also play an essential role in shaping wine quality and typicity. Particularly, anthocyanins are responsible for grape color, which is an important determinant of wine color. Grape anthocyanins derive from five anthocyanidins: cyanidin (Cy), delphinidin (Dp), peonidin (Pn), petunidin (Pt) and malvidin (Mv). They have different patterns of hydroxylation (di- or tri-hydroxylated forms), methylation, and can be further modified by acylation (Mazza, 1995). The fine-tuning of anthocyanin composition has important impacts on the color hue and color stability of the resultant wines (Mazza, 1995). Source-sink modulation impacts berry coloration (Weaver, 1963; Kliewer and Weaver, 1971; Petrie et al., 2000a), and recently its effects on anthocyanin content and composition (Guidoni et al., 2008; Pastore et al., 2011, 2013; Filippetti et al., 2015) drew attention of many research groups. For example, Wu et al. (2013) showed that retaining two leaves only in a girdled shoot with one cluster completely inhibited berry coloration. Moreover, Guidoni et al. (2008) reported that total anthocyanins were reduced by source limitation, with di-hydroxylated anthocyanins more sensitive than tri-hydroxylated ones in cv. Nebbiolo berries. However, other authors recently showed that a post-veraison source limitation resulting from either shoot trimming (Filippetti et al., 2015), removal of leaves above the clusters (Palliotti et al., 2013b; Poni et al., 2013) or late-season application of anti-transpirants (Palliotti et al., 2013a) significantly reduced the speed of sugar accumulation but did not affect the concentration of berry anthocyanins at harvest. As the source-sink modulation techniques also bring about concomitant modifications in the fruit zone microclimate (i.e., light and temperature regimes), the results must be interpreted with caution. It is well established that temperature significantly affects anthocyanin accumulation (Spayd et al., 2002; Pereira et al., 2006; Mori et al., 2007). Therefore, experiments that are more precisely controlled and avoid confounding between the effects of source-sink modulation and microclimate are needed to quantify the actual response of anthocyanins to carbon availability.

The accumulation of carbon in primary and secondary metabolites is interconnected and results from a complicated metabolic network. For instance, sugar levels positively correlate with total anthocyanin levels (Vitrac et al., 2000; Dai et al., 2014), yet negatively correlate with organic acids (Keller, 2010b). Interestingly, the accumulation of sugars and anthocyanins can be uncoupled by environmental conditions such as high temperature (Sadras and Moran, 2012). In contrast, the effect of source-sink modulation on the sugar-anthocyanin uncoupling seems more complicated (Sadras and Moran, 2012). The anthocyanin: sugar ratio has been reported to be increased (Guidoni et al., 2002), decreased (Sadras et al., 2007), or unchanged (Petrie and Clingeleffer, 2006) by increasing source-sink ratio. The mechanisms underlying this diversity of responses warrant further investigation. Several theories have been developed in literature to describe the relationship between primary and secondary metabolites in plants, and the two most relevant ones are the carbon-nutrient balance (CNB) hypothesis and the growth-differentiation balance (GDB) hypothesis (reviewed in Koricheva et al., 1998). CNB predicts that concentrations of carbon-based secondary metabolites will decrease in cases where carbon fixation is more reduced than growth, as a result of decreased available carbon pool for allocation to secondary metabolites production. GDB provides a similar prediction, and a meta-analysis showed that both hypothesis appears to be valid for describing the dependence of total C-based secondary metabolites, particularly phenylpropanoid-derived compounds (including anthocyanins) on carbon availability in the leaves of woody plant (Koricheva et al., 1998). If sugars and anthocyanins do have different sensitivities to source-sink modulation, such de-synchronization may help to define a window of source-sink ratios, within which sugars are reduced while anthocyanins are unaffected. This would provide valuable clues to mitigate the negative influences of climate change (Keller, 2010a).

Therefore, the present study aims to quantify the relative sensitivities of different berry compounds (sugars, organic acids, and anthocyanins) to changes in source-sink modulation under controlled or semi-controlled conditions. Monitoring the carbon fixation rate of the whole-canopy and dynamic profiling of metabolites allowed us to conduct a quantitative analysis of carbon demand and supply, and to obtain detailed information on the source-sink balance. In addition, a detailed HPLC analysis allowed us to compare the effects of source-sink modulation on the developmental changes in anthocyanin profiles in two distinct cultivars, Cabernet Sauvignon and Sangiovese.

## Materials and Methods

Two experiments were conducted with cv. Cabernet Sauvignon in Bordeaux (latitude 44^°^ 46^′^ 46^′′^ N, longitude 00^°;^ 34^′^ 01^′′^ W), France, and cv. Sangiovese in Piacenza (latitude 45^°;^02^′^52^′′^N, longitude 9^°;^42^′^2^′′^E), Italy.

### Plant Material and Sampling

#### Exp 1. Cabernet Sauvignon

Fruiting-cuttings made of one vertical shoot bearing one grape cluster of cv. Cabernet Sauvignon were prepared as described in Mullins and Rajasekaran (1981) and grown in a naturally lighted and semi-controlled greenhouse with chemical disease control applied every 2 weeks. Environmental conditions (air temperature, radiation at canopy level, and relative humidity) were recorded hourly throughout the experiment (Supplementary Figure S1).

Thirty homogeneous fruiting-cuttings were subjected to two source-to-sink ratios at 1 week before veraison; a group of 15 plants had 12 leaves per cluster per vine (12L) while the remaining vines had three leaves per cluster per vine (3L). At 63 days after flowering (DAF), leaves underneath the basal cluster were removed in both treatments to standardize the microclimate effects; therefore, above the cluster, 3 and 12 leaves were maintained, yielding a total of 7 and 16 nodes per shoot for the 3L and 12L treatments, respectively. The remaining leaves and all secondary shoots were removed over the measurement period. The plants were randomly assigned to three blocks and each block composed of five plants of each treatment.

Berries were sampled nine times at 1-week interval from 1 week after treatment (70 DAF) to 126 DAF. In order to ensure the capture of maturity in 3L treated vines, the last sampling date corresponded to an over-ripe stage. At each sampling date, one berry each was sampled from the top and the middle of a single cluster, and the resulting 10 berries from five clusters (vines) of a given treatment within a plot were pooled to form a biological replicate. Three biological replicates were obtained for each treatment at each sampling date. At harvest, all remaining berries were sampled, counted, and weighed.

#### Exp 2. Sangiovese

The experiment was conducted on 4-years-old cane-pruned cv. Sangiovese grapevines grafted on M3 rootstock and grown outdoors in 40 L pots. The pots were filled with a mixture of sand, loam and clay (65, 20, and 15% by volume, respectively) and kept well watered throughout the trial season. Each vine had a 1 m long fruiting cane with 8–9 dormant buds. Shoot thinning was applied to retain one main shoot per node and, on each shoot, the basal cluster only was maintained. Vines were arranged along a single, vertically shoot-positioned, 35^°;^NE-SW oriented row and hedgerow-trained. Eight uniform vines were assigned in a completely randomized design to the following two treatments 1 week before veraison: three leaves per cluster (shoot) or 12 leaves per cluster (shoot). As in the Cabernet Sauvignon experiment, at 40 DAF, leaves beneath the basal cluster were removed in both treatments to standardize the microclimate effects; therefore, above the cluster, 3 and 12 leaves were maintained, for 3L and 12L treatment, respectively. Shoots were trimmed to 8 and 16 nodes per shoot, for 3L and 12L treatment, respectively. The remaining leaves and all secondary shoots were removed throughout the measuring period.

Berries were sampled 14 times at 1-week interval from 1 week before treatment to 8 weeks after treatment, and thereafter at 4-days intervals for better capturing maturity. At each sampling date, three berries from the top and the middle of a single cluster were sampled and the 24 or 27 berries from 8 or 9 clusters (shoot) of a given vine under a treatment were pooled to form a biological replicate. Four biological replicates were obtained for each treatment at each sampling date. At harvest, all remaining berries of a vine were sampled, counted, and weighed.

### Berry Pretreatment

Sampled berries from both experiments were immediately put into a pre-weighed tube and dropped into liquid nitrogen. The tubes were reweighed after deep freeze to calculate berry fresh weight and then stored in -80^°;^C for later biochemical analysis. Berries stored in -80^°;^C were slightly thawed and separated quickly into skin, pulp, and seed in the laboratory. The skin and pulp were immediately ground into fine powder in liquid nitrogen using a ball grinder MM200 (Retsch, Haan, Germany).

### Sugars and Organic Acids

An aliquot of 500 mg fine powder of pulp was extracted sequentially with ethanol (80 and 50%), dried in Speed-Vac, and re-dissolved in 2.5 mL de-ionized water. Glucose and fructose content were measured enzymatically with an automated micro-plate reader (Elx800UV, Biotek Instruments Inc., Winooski, VT, USA) according to the method of Gomez et al. (2007). Tartaric acid content was assessed by using the colorimetric method based on ammonium vanadate reactions (Pereira et al., 2006). Malic acid was determined using an enzyme-coupled spectrophotometric method that measures the change in absorbance at 340 nm from the reduction of NAD+ to NADH (Pereira et al., 2006).

### Analysis of Anthocyanins

An aliquot of 500 mg of berry skin powder was freeze-dried for 72 h and the dried powder (∼50 mg) were extracted in 1.0 mL methanol containing 0.1% HCL (v/v). Extracts were filtered through a 0.45 μm polypropylene syringe filter (Pall Gelman Corp., Ann Harbor, MI, USA) for HPLC analysis. Each individual anthocyanin was analyzed as described in Hilbert et al. (2003) and Acevedo De la Cruz et al. (2012) with HPLC. Quantification was carried out by peak area integration at 520 nm, and Malvidin-3-glucoside (Extrasynthèse, Lyon, France) standard was used for quantify the anthocyanin concentration.

### Leaf Area Measurement

For Cabernet Sauvignon experiment, leaf area (LA) was estimated using the relationship between specific LA (m^2^ fresh area gDW^-1^) and total leaf dry weight as described in Castelan-Estrada et al. (2002). LAs of the removed leaves for 3L treatment were determined at the initiation of treatment and whole plant LAs were determined for both treatments at the end of the experiment.

For Sangiovese experiment, LA was measured on the leaves that were removed the day of treatment and after harvest of all berries. LA was determined by measuring the surface of each lamina with a LA meter (LI-3000A, LI-COR Biosciences, Lincoln, NE, USA).

### Chlorophyll Concentration

Six leaves per plant for 12L vines and three leaves per plant for 3L vines were measured using the portable Chlorophyll Meter SPAD 502 (Minolta Corp., Ramsey, NJ, USA). On each leaf, five SPAD readings were taken at each leaf lobe and then averaged.

### Single-Leaf Gas Exchange

Net photosynthesis (P_n_), evapotranspiration (E) and stomatal conductance (g_s_) rates of six leaves per plant were measured only in the Sangiovese experiment at 95 DAF using a CIRAS-2 portable photosynthesis system (PP Systems, Amesbury, MA, USA). On each vine, two shoots were chosen in basal and apical positions along the cane and, on each shoot, three mature leaves located in the basal, median, and apical positions of the main stem were measured in rapid sequence. Readings were performed in the morning hours (10 h 00–12 h 00) under constant saturating light (≈1500 μmol m^-2^ s^-1^) imposed with an additional external lamp mounted on top of the leaf chamber. Measurements were taken at ambient relative humidity and the flow fed to the broad-leaf chamber (4.5 cm^2^ window size) was 300 mL min^-1^. To ensure stability of the inlet reference CO_2_ concentration [CO_2_], a CO_2_ minicartridge was used to provide automatic control of inlet [CO_2_] at 380 mmol L^-1^.

### Whole-Canopy Gas Exchange

Whole-canopy net CO_2_ exchange rate (NCER) measurements were performed only in the Sangiovese experiment using the multi-chamber system reported in Poni et al. (2014) featuring alternating current, centrifugal blowers (Vorticent C25/2M Vortice, Milan, Italy) delivering a maximum air flow of 950 m^3^ h^-1^; flexible plastic polyethylene chambers allowing 88% light transmission, 6% diffuse light enrichment and no alteration of the light spectrum. System also features a CIRAS-EGM4 single-channel absolute CO_2_ infrared gas analyser (PP Systems, Amesbury, MA, USA) set at a 0–1000 parts per million measurement range and a CR1000 data logger wired to an AM16/ 32B Multiplexer (Campbell Scientific, Shepshed, UK). To facilitate air mixing and ensure higher stability in inlet CO_2_ concentration, air was forced through a buffer tank (500 L) before being directed to the chambers. Switching of air sampling from one chamber to another was achieved at programmed time intervals (90 s) using a set of solenoid valves (SIRAI, Padova, Italy); the air-flow rate to each chamber was controlled by a butterfly valve (Ghibson, Monteveglio, Italy) and measured with a Testo 510 digital manometer (Farnell, Lainate, Italy) using the flow restriction method (Osborne, 1977). The flow rate fed to the chambers was set at 7.1 L/s and kept until the leaf removal treatment, when the flow rate was changed to 5 L/s. Whole-canopy NCER per vine (μmol CO_2_/s) and per LA unit (μmol CO_2_/m^2^s) was calculated from flow rates and CO_2_ differentials after.

The chambers were set up on each vine and continuously operated 24 h per day from one week before treatment (2 July) until 95 days after flowering (1 September). Ambient (inlet) air temperature and the air temperature at each chamber’s outlet were measured by shielded 1–0.2 mm diameter PFA–Teflon insulated type-T thermocouples (Omega Engineering, Stamford, CT, USA), and direct and diffuse radiation were measured with a BF2 sunshine sensor (Delta-T Devices, Ltd, Cambridge, England) placed horizontally on top of a support stake next to the chambers enclosing the canopies. Ambient (inlet) relative humidity and the relative humidity at each chamber’s outlet were measured by a HIH-4000 humidity sensor (Honeywell, Freeport, IL, USA) mounted upstream of the EGM4.

### Data Analysis

All data analysis were conducted with R software (R Development Core Team, 2010). Student *t*-test was used to verify the differences between the two source-sink ratios at each developmental stage.

Carbon allocation analysis was conducted as it follows. First, the carbon accumulated in berries throughout development was calculated as a function of hexose concentration and berry fresh weight with a carbon transformation coefficient of 0.4 g carbon g^-1^ hexose. Second, the total carbon accumulated (C) in berries per vine was fitted to the following sigmoid curve (Sadras et al., 2008):

$$C\;=\;\frac{C_{max}}{1\;+\;e^{\left[\frac{t_{0}-t}{b}\right]}}$$

where *t* is the number of days after flowering, C_max_ is the maximal quantity of carbon (g), t_0_ is the number days after flowering when carbon quantity is half the maximum, and b represents the carbon accumulation duration from 0.25 C_max_ to 0.75 C_max_. Third, the carbon accumulation rate (g Carbon/day) was calculated by using the first order derivation of the sigmoid curve. Finally, the relationship between the berry accumulated carbon and the photosynthesized carbon (obtained from whole-canopy gas exchange measurement) was quantified by their ratio to estimate the supply vs. demand carbon balance.

## Results

### Leaf-to-Fruit Ratio

As expected, leaf removal effectively reduced the total LA per vine in 3L treatment in Cabernet Sauvignon and Sangiovese (**Table 1** and **2**). It also resulted in a significantly lower leaf-to-fruit ratio (LA/F) in 3L than 12L treatments in both cultivars. 3L vines all had a LA to yield ratio lower than 1.0 m^2^/Kg. On the other hand, both 12L Cabernet Sauvignon vines and Sangiovese vines had a LA/F of 3.98 m^2^/Kg and 1.15 m^2^/kg, respectively (**Table 1** and **2**).

**Table 1:** Effect of source-sink modulation on leaf area (LA), leaf area-to-yield ratio, and leaf chlorophyll content (SPAD) of Cabernet Sauvignon grapevines.

Treatment^‡^	Pre-trimming LA/vine (cm^2^)	Removed LA/vine (cm^2^)	Final LA/vine (cm^2^)	LA/yield (m^2^/Kg)	SPAD
3L	1123	858	265	0.67	51.0
12L	999	0	999	3.98	44.1
Sig.(*t* test)^+^	ns	^∗∗^	^∗∗^	^∗∗^	^∗∗^

*Data are means of nine plants. ^‡^3L: plants with three leaves per cluster; 12L: plants with twelve leaves per cluster. ^+^, ^∗∗^, and ns indicate statistical significance at *P* = 0.001, and not significant, respectively*.

**Table 2:** Effect of source-sink modulation on leaf area (LA), whole net carbon exchange rate (NCER) per vine and per unit of leaf area, cumulated net carbon fixation (gCO_2_/vine) over the trial period, and leaf-to-yield ratio of Sangiovese grapevines.

Treatment^‡^	Pre-trimming LA (*m*^2^)	Removed LA (*m*^2^)	Final LA (*m*^2^)	NCER/vine (μmols^-1^)		NCER/LA (μmolm^-2^s^-1^)		gCO_2_/vine (cumulated over trial period)	LA/yield (m^2^/Kg)
				Pre	Post	Pre	Post		
3L	1.86	1.55	0.31	8.19	1.94	4.39	6.30	142	0.33
12L	1.70	0.68	1.02	8.04	5.64	4.67	5.56	321	1.15
Sig. (*t*-test)^+^	ns	^∗∗^	^∗∗^	ns	^∗∗^	ns	ns	^∗∗^	^∗∗^

*Data are means of four plants. NCER/vine and NCER/LA were averaged for all the post-treatment measurements. ^‡^3L: plants with three leaves per cluster; 12L: plants with 12 leaves per cluster ^+^, ^∗∗^, and ns indicate statistical significance at *P* = 0.001, and not significant, respectively*.

### Berry Weight

**FIGURE 1:**
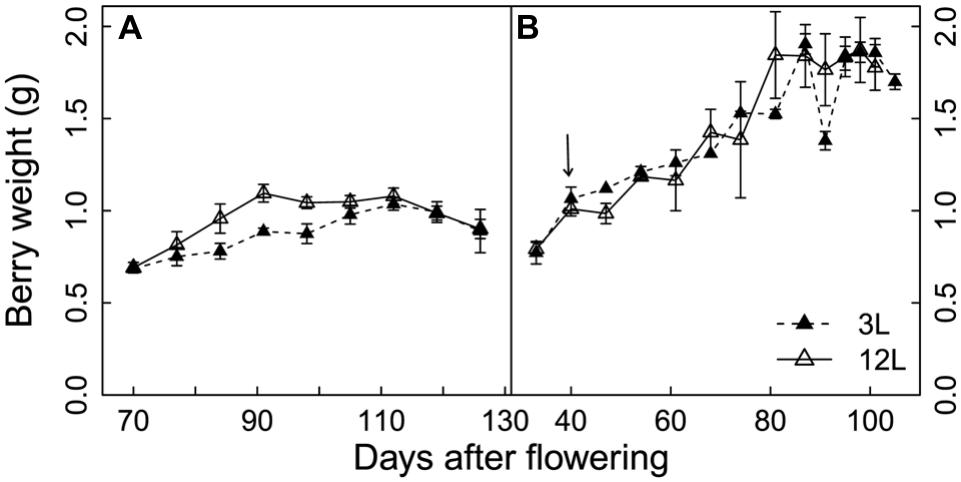
**Effect of source-sink modulation on seasonal berry weight of Cabernet Sauvignon **(A)** and Sangiovese **(B)** vines having either three leaves (3L) or 12 leaves per cluster (12L)**. The solid arrow indicates date of source-sink modulation. Vertical bars indicate SE (*n* = 3 for Cabernet Sauvignon, and *n* = 4 for Sangiovese).

In Cabernet Sauvignon, the 3L treatment limited the increase in berry size that normally occurs between 80 and 90 days after flowering (DAF), but extended the growth duration to 110 DAF, when berries under 12L conditions already reached their maximal fresh weights (**Figure 1A**). The longer growth duration compensated for the decreased growth rate and resulted in a very similar berry weight under both growth conditions at harvest. Conversely, 3L treatment did not affect the developmental profile of berry size in Sangiovese berries (**Figure 1B**). At harvest, Sangiovese berries were bigger than those of Cabernet Sauvignon. Berries of both cultivars doubled their size from veraison to maturity. In addition, berry dehydration occurred in Cabernet Sauvignon berries as indicated by the decrease in berry fresh weight from 112 DAF to 126 DAF (**Figure 1A**).

### Sugar Concentration

**FIGURE 2:**
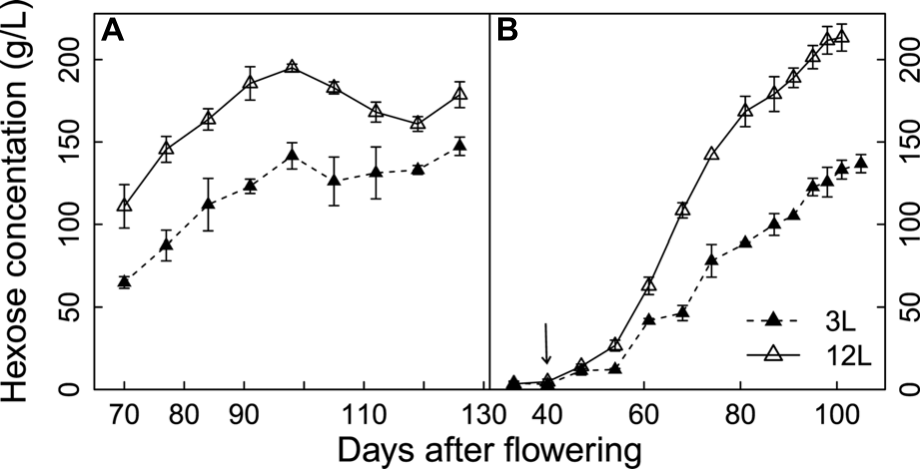
**Effect of source-sink modulation on seasonal hexose concentrations of Cabernet Sauvignon **(A)** and Sangiovese **(B)** berries sampled from vines having either three leaves (3L) or 12 leaves per cluster (12L)**. The solid arrow indicates date of source-sink modulation. Vertical bars indicate SE (*n* = 3 for Cabernet Sauvignon, and *n* = 4 for Sangiovese).

Hexose (glucose + fructose) concentrations of Cabernet Sauvignon and Sangiovese berries were significantly reduced by source limitation (**Figure 2**). The negative effects of source limitation were observed 1 week after treatment for Cabernet Sauvignon and 2 weeks after treatments for Sangiovese. At harvest, 3L treatment caused a 17.5% reduction of hexose concentration in Cabernet Sauvignon and a 36.7% reduction in Sangiovese compared to 12L treated berries.

### Organic Acids

**FIGURE 3:**
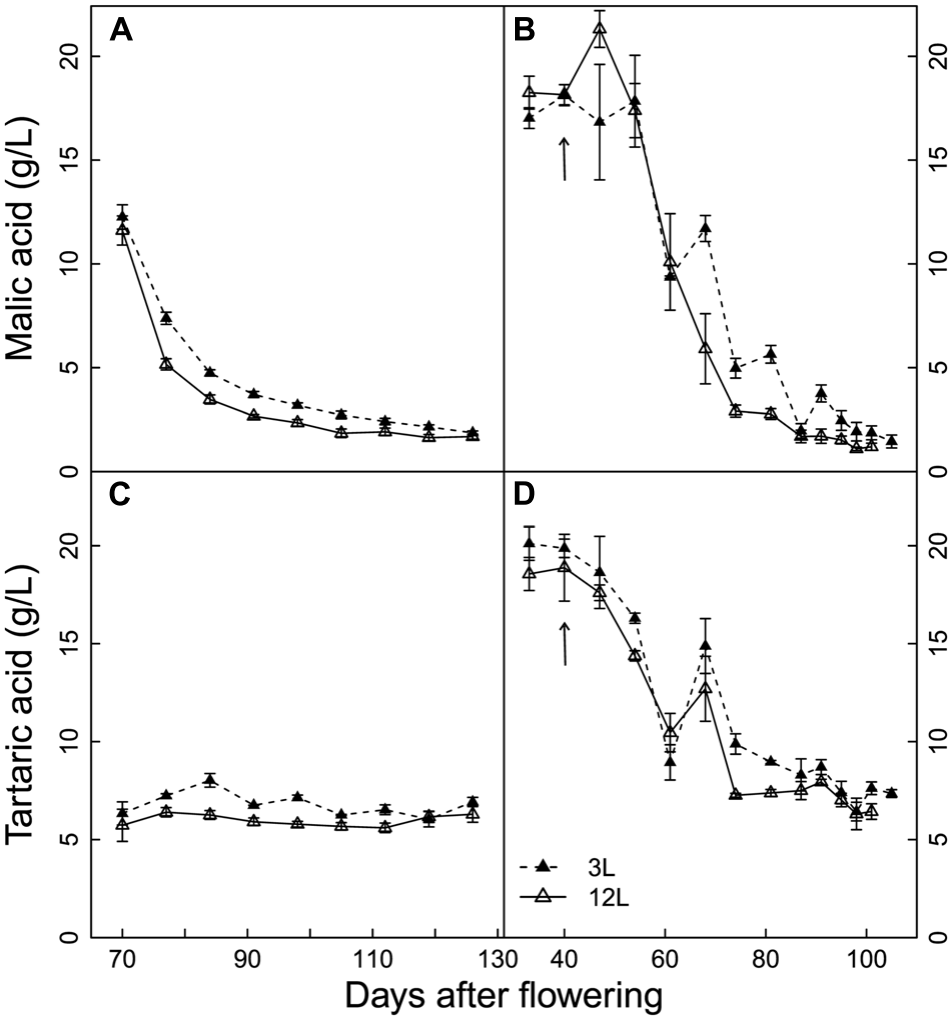
**Effect of source-sink modulation on seasonal malic acid **(A,B)** and tartaric acid **(C,D)** concentration of Cabernet Sauvignon **(A)** and Sangiovese **(B)** berries from vines having either three leaves (3L) or 12 leaves per cluster (12L)**. The solid arrows indicate date of source-sink modulation. Vertical bars indicate SE (*n* = 3 for Cabernet Sauvignon, and *n* = 4 for Sangiovese).

The developmental profiles of malic and tartaric acids were slightly affected by the source-sink modulation in Cabernet Sauvignon berries (**Figures 3A,C**). From veraison to near harvest, the concentrations of malic and tartaric acids were higher in the 3L than in the 12L treatment, while no significant differences were found at harvest. On the other hand, the developmental profiles of organic acids in Sangiovese berries were not significantly affected by source-sink modulation (**Figures 3B,D**). In addition, the flat trend of tartaric acids in Cabernet Sauvignon berries is due to the fact that sampling started at a later stage (**Figure 3C**). At harvest, the concentrations of malic acid were 1.78 and 1.5 g/L and those of tartaric acids were 6.61 and 7.13 g/L for Cabernet Sauvignon and Sangiovese, respectively.

### Anthocyanin Composition and Concentration

**FIGURE 4:**
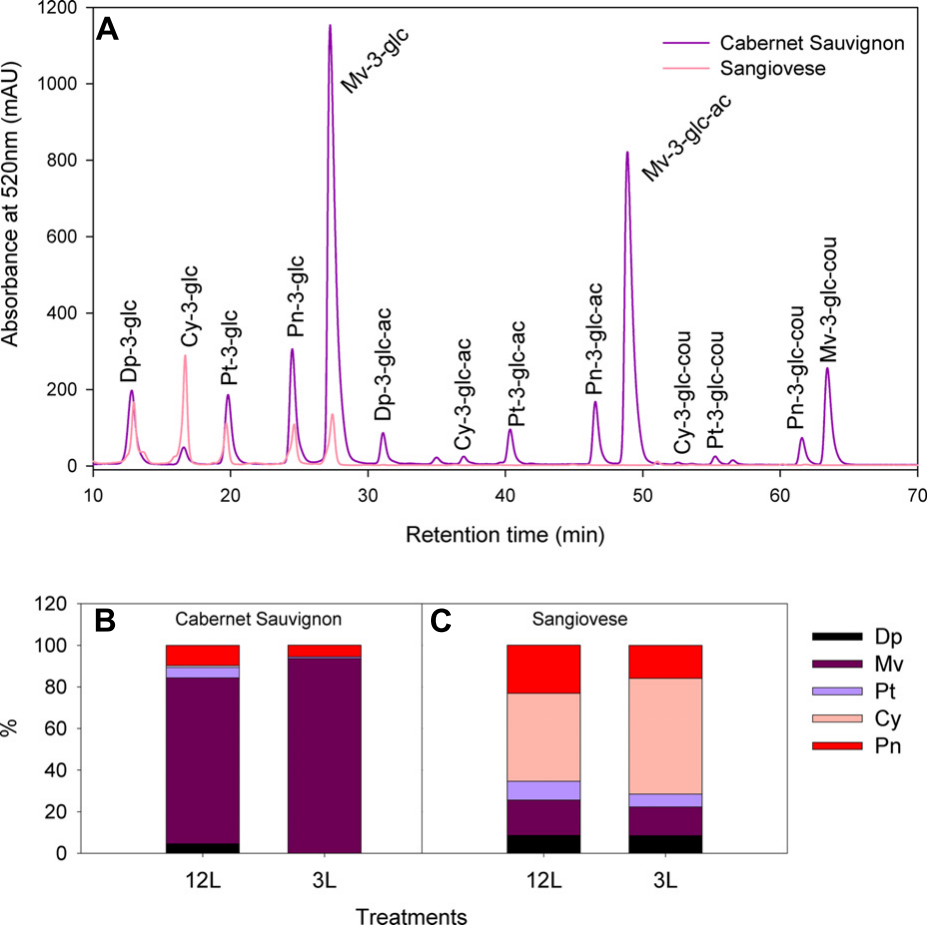
**Typical HPLC chromatograms of anthocyanins in berry skins of Cabernet Sauvignon and Sangiovese grape berries grown under non-limited carbon supply **(A)**; and effects of source-sink modulation on anthocyanin composition of Cabernet Sauvignon **(B)** and Sangiovese **(C)** berry skins at harvest from vines with either three leaves (3L) or 12 leaves per cluster (12L)**. Dp, delphinidin; Mv, malvidin; Pt, petunidin; Cy, cyanidine; Pn, peonidin; glc, glucoside; ac, acetate; cou, coumarate.

Cabernet Sauvignon and Sangiovese berries showed different anthocyanin profiles under adequate source supply (**Figure 4**), with malvidin-derivatives dominant in the former and cyanidine-3-glucoside dominant in the latter. Moreover, all the acylated forms of anthocyanins were absent in Sangiovese (**Figure 4A**). These differences in composition were further affected by source limitation (**Figures 4B,C**). At harvest, the proportion of malvidin-derivatives was increased to 93.7% in 3L-treated berries in comparison with 79.7% in 12L-treated berries of Cabernet Sauvignon (**Figure 4B**). Sangiovese was less affected by source limitation, with 55.6% of cynidin-3-glucoside in 3L-treated berries and 42.4% in 12L-treated berries (**Figure 4C**).

**FIGURE 5:**
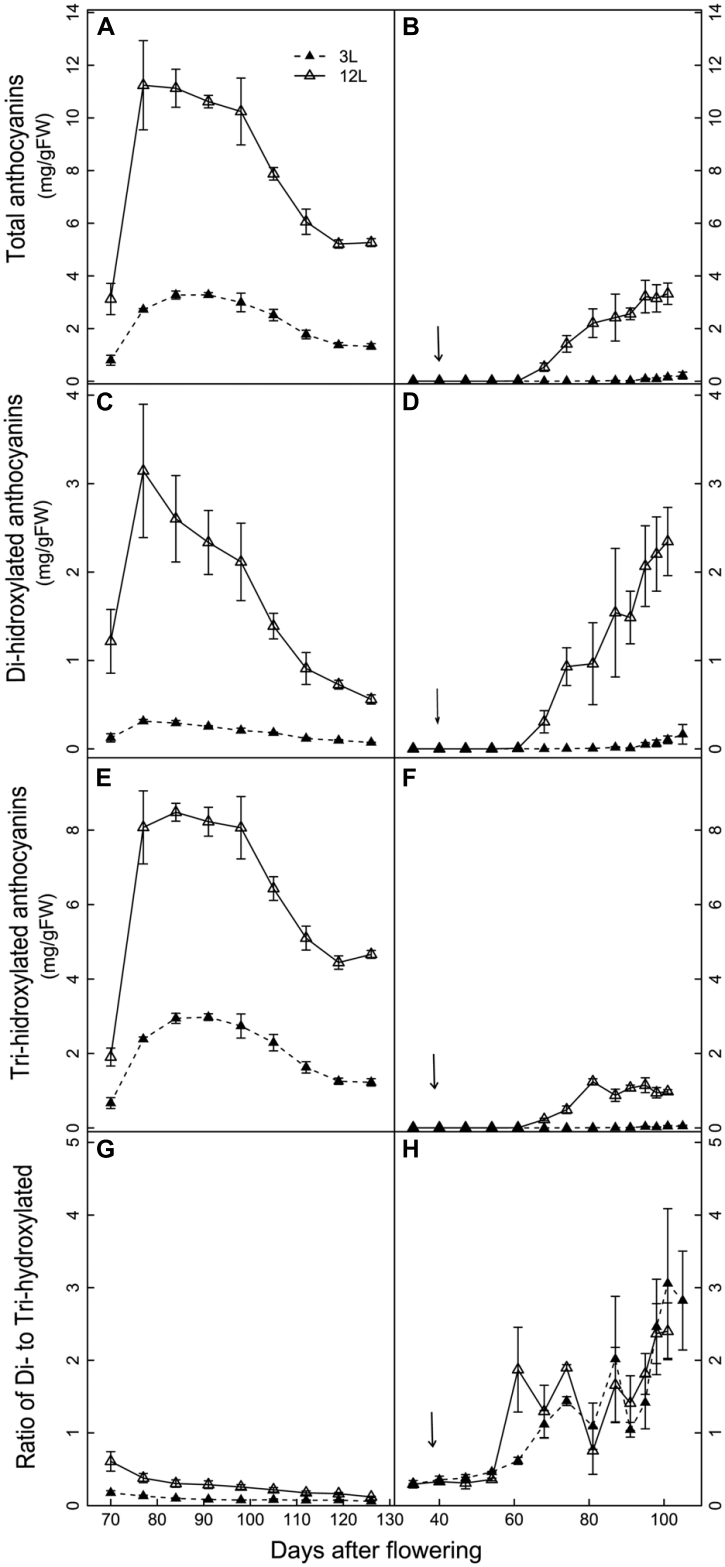
**Effect of source-sink modulation on the accumulation of total anthocyanins **(A,B)**, di-hydroxylated anthocyanins **(C,D)**, tri-hydroxylated anthocyanins **(E,F)**, and the ratio of Di- to Tri-hydroxylated anthocyanins **(G,H)** in the skin of Cabernet Sauvignon (left panel) and Sangiovese berries (right panel)**. The two carbon supply levels were obtained by treating vines with either three leaves (3L) or 12 leaves per cluster (12L). The solid arrows indicate date of source-sink modulation. Vertical bars indicate SE (*n* = 3 for Cabernet Sauvignon, and *n* = 4 for Sangiovese).

**FIGURE 6:**
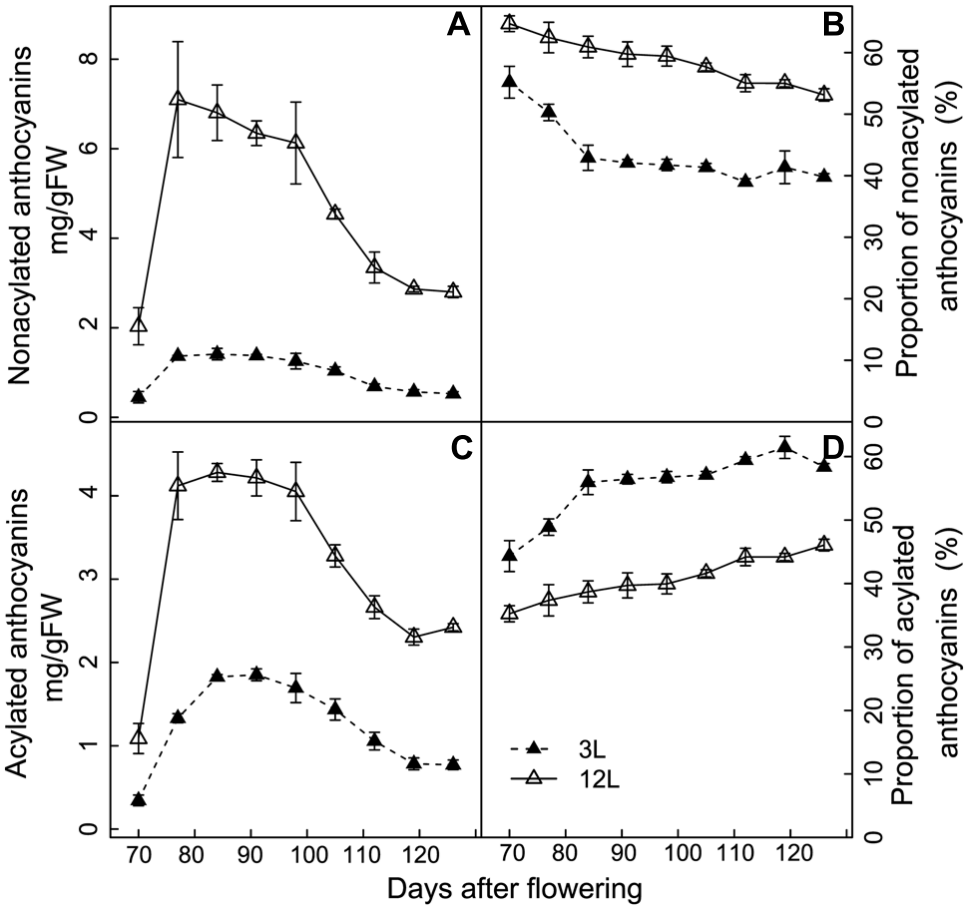
**Effect of source-sink modulation on the quantity **(A,C)** and proportion **(B,D)** of non-acylated anthocyanins **(A,B)** and acylated anthocyanins **(C,D)** in relation to the total concentration in the skin of Cabernet Sauvignon berries from vines with either three leaves (3L) or 12 leaves per cluster (12L)**. Vertical bars indicate SE (*n* = 3).

The developmental profiles of anthocyanin composition and concentration were significantly affected by source limitation in both cultivars (**Figures 5** and **6**). In Cabernet Sauvignon, the amount of total anthocyanins was systematically higher in 12L treated berries than in 3L treated berries throughout berry development (**Figure 5A**). 12L treated berries increased their anthocyanins sharply from 70 to 77 DAF, remained at a plateau until 98 DAF, and thereafter decreased progressively. 3L berries exhibited a similar developmental profile but with a much lower plateau than 12L berries. In Sangiovese, 12L berries started to accumulate anthocyanins from 68 DAF and reached a maximum at harvest. By contrast, 3L treatment almost completely depressed the accumulation of total anthocyanins, with only a slight increase between 95 to 105 DAF (**Figure 5B**). At harvest, 3L treatment caused a 74.8% reduction in the concentration of total anthocyanins in Cabernet Sauvignon (1.32 mg/g FW in 3L versus 5.27 mg/g FW in 12L) and a 94.5% reduction in Sangiovese (0.22 mg/gFW in 3L versus 3.32 mg/gFW in 12L).

Tri-hydroxylated and di-hydroxylated anthocyanins showed different developmental profiles and distinct responses to source limitation (**Figures 5C–H**). Cabernet Sauvignon berries had higher tri-hydroxylated anthocyanins than di-hydroxylated ones in both treatments (**Figure 5C,E,G**), while the reverse was observed in Sangiovese (**Figures 5D,F,H**). In Cabernet Sauvignon, 3L treatment decreased more di-hydroxylated (**Figure 5C**) than tri-hydroxylated anthocyanins (**Figure 5F**), resulting in a lower ratio of di- to tri-hydroxylated anthocyanins (**Figure 5G**). By contrast, 3L treatment equally decreased both the di- and tri-hydroxylated anthocyanins in Sangiovese, leaving the ratio of di- to tri-hydroxylated anthocyanins unaffected (**Figure 5H**).

The effect of source-sink modulation on anthocyanin acylation was further investigated in Cabernet Sauvignon berries (**Figure 6**). 3L treatment decreased more strongly the non-acylated anthocyanins than the acylated ones in comparison with 12L treated berries (**Figures 6A,B**). This unbalanced modification caused a significant increase in the proportion of acylated anthocyanins in 3L treated berries than 12L treated berries (**Figure 6D**). The proportion of acylated anthocyanins reached 58.4% in 3L and was 46.1% in 12L at harvest (**Figure 6D**).

### Leaf Carbon Fixation and Berry Carbon Utilization

**FIGURE 7:**
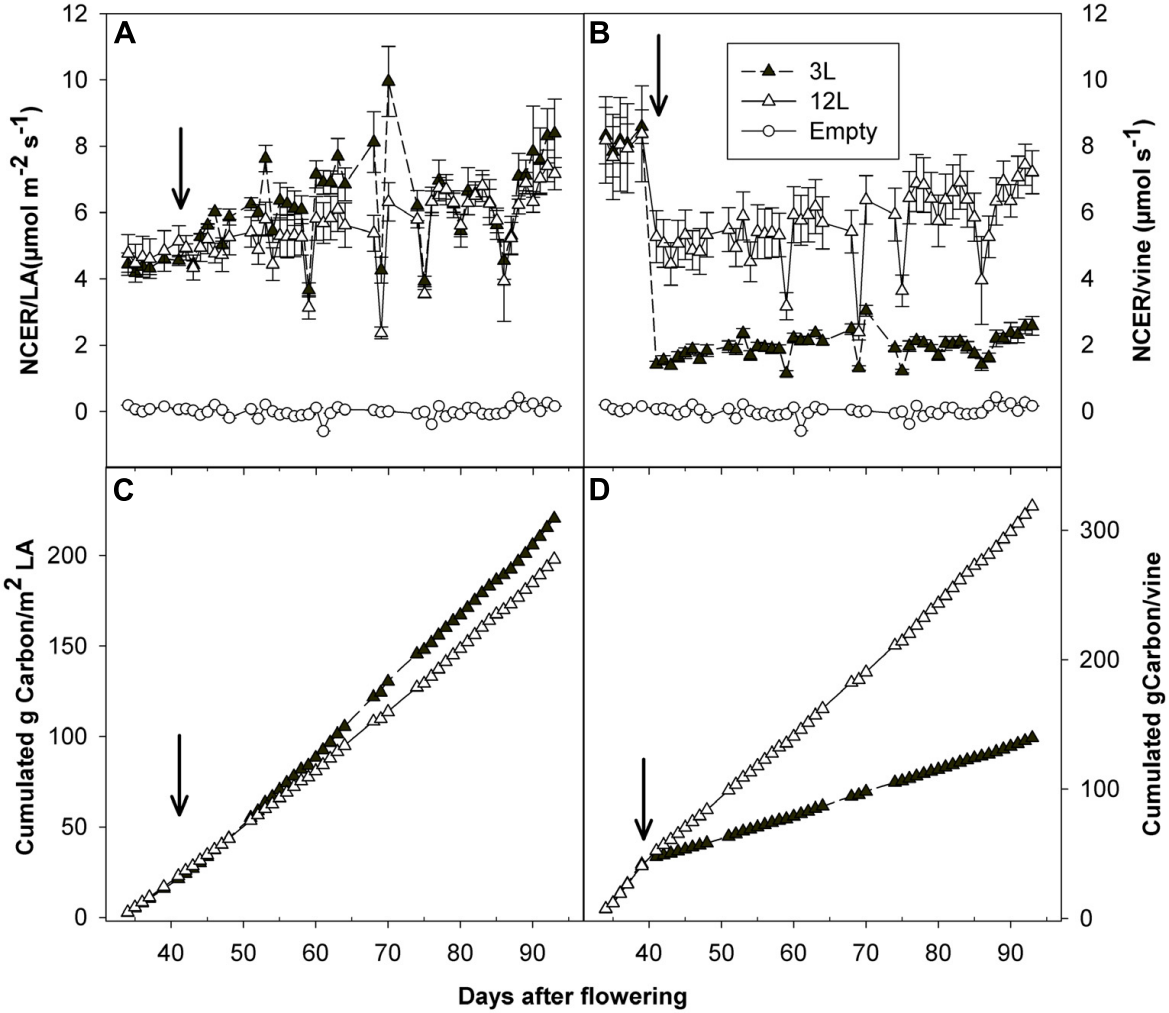
**Effect of source-sink modulation on net carbon exchange rate (NCER) per unit of leaf area **(A)** and per vine **(B)**, as well as cumulated carbon fixation per unit of leaf area **(C)** and per vine **(D)**, recorded daily with a whole-canopy gas exchange system throughout the experimental period on Sangiovese vines either with three leaves (3L) or 12 leaves per cluster (12L)**. Values from empty chambers were also indicated **(A,B)** as a reference. The solid arrows indicate date of source-sink modulation. Vertical bars indicate SE (*n* = 4).

Whole-canopy net CO_2_ exchange rates (NCER) were measured in Sangiovese and are shown in **Figure 7**. The NCER per unit LA (μmol m^-2^ s^-1^) was very similar (≅ 4.5 μmol m^-2^ s^-1^) for the two groups of vines before treatment (**Figure 7A** and **Table 2**). After treatment, it became slightly higher in 3L (in average 6.30 μmol m^-2^ ^-1^) than in 12L treatment (in average 5.56 μmol m^-2^ s^-1^), without reaching a significant difference though (**Table 2**). However, when those marginal differences in each day were cumulated, the carbon fixed by a unit of LA over the experimental period was clearly higher in 3L than 12L treatment (**Figure 7C**). Single leaf photosynthesis rate at harvest, measured under optimal conditions at saturating light, was significantly higher in 3L treated leaves (15.1 μmol m^-2^ s^-1^ ) than in 12L treatment (13.8 μmol m^-2^ s^-1^). Moreover, for both Cabernet Sauvignon (**Table 1**) and Sangiovese (**Table 3**), leaves from 3L plants had higher chlorophyll content than that of 12L plants (**Table 3**).

**Table 3:** Effect of source-sink modulation on transpiration (E), stomatal conductance (g_s_), net photosynthesis (Pn) and chlorophyll content (SPAD) measured on six leaves per vine in Sangiovese.

Treatment^‡^	E (mmol m^-2^ s^-1^)	g_s_ (mol m^-2^ s^-1^)	P_n_ (μmol m^-2^ s^-1^)	SPAD
3L	8.59	0.368	15.1	40.9
12L	8.86	0.345	13.8	38.0
Sig.(*t*-test)^+^	ns	ns	^∗∗^	^∗^

*Data are means of four plants. ^‡^3L: plants with three leaves per cluster; 12L: plants with 12 leaves per cluster. ^+^^∗^,^∗∗^, and ns indicate statistical significance at *P = 0.05, 0.001*, and not significant, respectively*.

The NCER per vine was calculated as the product of NCER per LA and total LA per vine (**Figure 7B**). Before treatment, leaves of both groups had very similar NCER/vine (average of 3L and 12L at 8.12 μmol s^-1^). During the treatment, 40 and 83.3% LA were removed in comparison with pre-treatment for 12L and 3L vines, respectively (**Table 2**). These reductions in total LA per vine resulted in an abrupt proportional decrease of NCER/vine of 38.8% for 12L vines and 82.4% for 3L vines, averaged during the first 3 days after treatment (**Figure 7B**). Thereafter, vines reacted to their treatments, and the 3L plants showed a 66.1% reduction in NCER/vine in parallel with a 69.6% reduction in LA per vine, as compared to 12L plants (**Figure 7B** and **Table 2**). When the NCER per vine was cumulated over the post-treatment period, the cumulated carbon per vine at harvest in 3L treatment was only reduced by 55.8% compared to 12L (**Figure 7D**).

**FIGURE 8:**
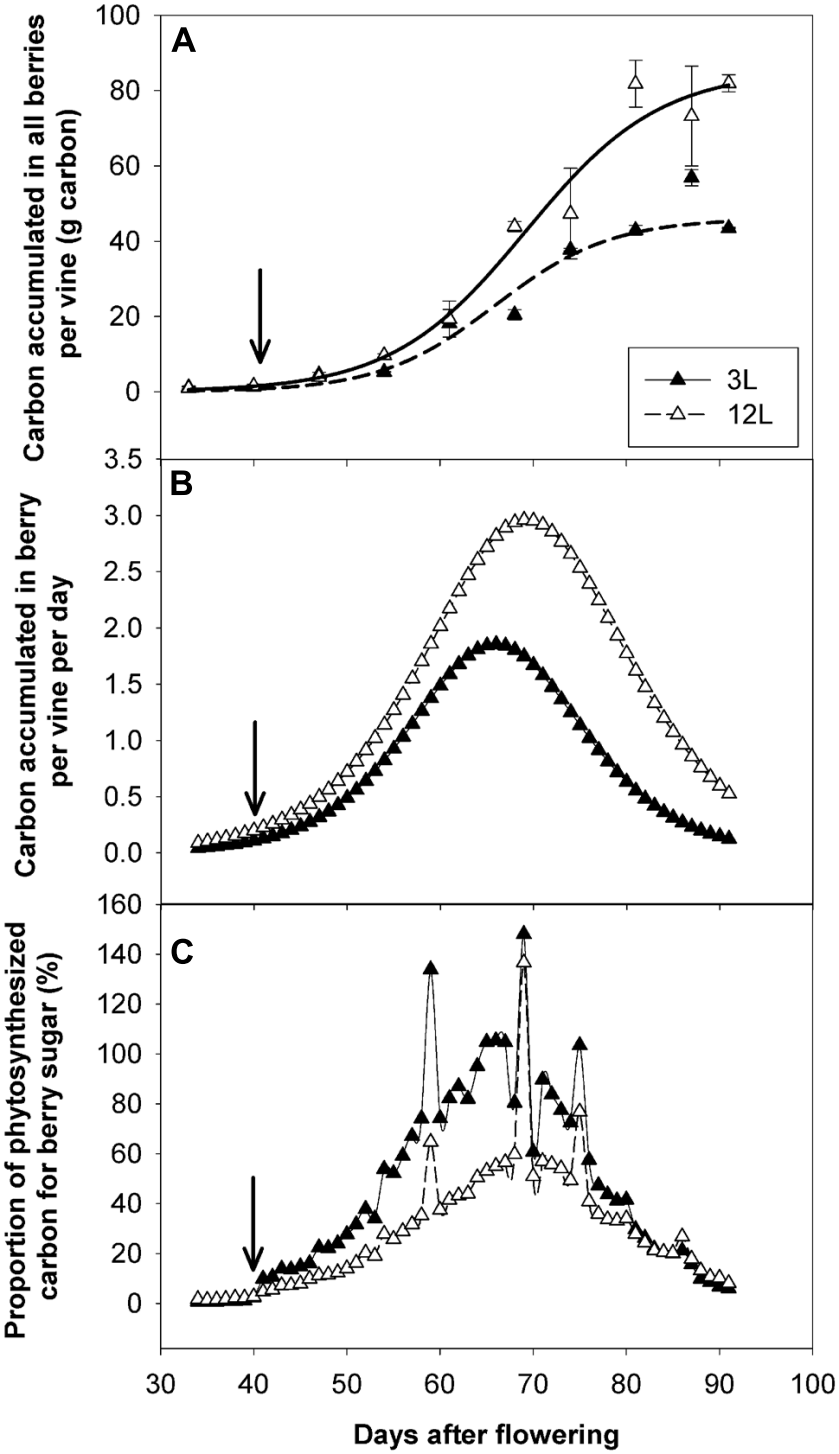
**Effect of source-sink modulation on carbon allocation in Sangiovese grown with three leaves (3L) or 12 leaves (12L) per cluster**. Carbon accumulated in all berries per vine **(A)**, carbon accumulation rate in all berries per vine per day **(B)**, and proportion of photosynthesized carbon used for berry sugar accumulation during the developmental period **(C)**. The solid arrows indicate date of source-sink modulation.

To investigate the carbon supply-demand balance, carbon accumulation rate in all the berries of a vine was also calculated and compared with the carbon fixed by photosynthesis (**Figure 8**). As expected, 3L berries accumulated much lower carbon (43.4 g) than those of 12L treatment (81.9 g) at harvest (**Figure 8A**). In parallel, the carbon accumulation rate in berries (g carbon per day) was also decreased in 3L berries compared to 12L berries (**Figure 8B**). Considering the carbon accumulation rate as carbon utilization and the NCER per vine as carbon supply, the proportion of the former to the latter was calculated (**Figure 8C**). Total accumulated carbon per day in 3L berries accounted for ∼76.9% of that fixed by photosynthesis during the rapid sugar accumulation period (namely from 54 to 81 DAF), while it accounted for only ∼48% in 12L berries over the same period. Interestingly, this proportion jumped to very high levels, even more than 100%, in several specific days in both treatments. Further analysis revealed that those days corresponded to cloudy days (Supplementary Figure S1) when NCERs per vine were very low (**Figure 7B**).

## Discussion

Source limitation induced by severe leaf removal in Cabernet Sauvignon and Sangiovese caused a significant reduction in the accumulation of sugars in berries. This result confirms many previous studies with different other *Vitis vinifera* L. cultivars, that reported a decrease in sugar accumulation following carbon limitation induced by late leaf removal (manual or mechanical), shoot trimming at fruit set and veraison (Poni and Giachino, 2000; Heuvel et al., 2005; Stoll et al., 2010; Palliotti et al., 2013b; Poni et al., 2013; Filippetti et al., 2015; Parker et al., 2015). However, there are also studies conversely showing that sugar accumulation is unaffected (Percival et al., 1994; Chorti et al., 2010; Sabbatini and Stanley Howell, 2010; Pastore et al., 2013) or even slightly increased (Bledsoe et al., 1988; Percival et al., 1994; Guidoni et al., 2002; Poni et al., 2006a, 2008; Palliotti et al., 2011, 2012; Pastore et al., 2011, 2013; Bubola and Persuric, 2012; Gatti et al., 2012) after a diminishing LA/Fruit ratio. These contradictory observations are most likely the result of the differences in the timing and severity of source to sink modulations. In fact, Kliewer and Dokoozlian (2005) have shown that a LA/Fruit above 0.8 m^2^/Kg is critical for full ripening of the grapes. Studies reporting no effect of source-sink modulations on sugar accumulation often did not go below this threshold value. In the present study, the LA/Fruit was lower than 0.8 m^2^/Kg in source limited vines for both cultivars. It is therefore of the utmost importance to consider the magnitude of LA/Fruit when compare trials on the effect of source-sink modulations on berry sugars.

In contrast to the decrease in sugar concentration under source limitation, no significant differences were found in organic acids content at harvest in the two cultivars studied in this work. In a recent detailed developmental analysis, Parker et al. (2015) also observed lose of synchronization between sugar and organic acid in response to lowering LA/Fruit ratios, with sugar accumulation reduced but organic acids largely unaffected. Some other studies (Bledsoe et al., 1988; Poni et al., 2006a, 2009; Tardaguila et al., 2010; Pastore et al., 2013) reported that a decrease in source-sink ratio by leaf removal lead to decreased total acidity and malic acid, whereas tartaric acids was unaffected or even increased compared to vines without leaf removal. The authors of these studies frequently pointed out a likely confounding effect of leaf removal and modified microclimate, making their result not really comparable with those found in the present study. Our results and those from Parker et al. (2015) both confirm that the organic acids are less responsive to carbon limitation than sugars.

In addition to the relative sensitivity of sugars and organic acids, we also studied the response of important secondary metabolites such as anthocyanins. Source limitation caused 75 and 93.5% reductions in anthocyanin concentrations in Cabernet Sauvignon and Sangiovese berries at harvest, paralleled by only 17.5 and 36.7% reductions in sugars, as compared to the non-source limited berries. After recalculation from the dataset of recent reports, we found that source limitation caused a 99.2% reduction in anthocyanins with a 38.5% reduction in sugars in cv. Jingyan (*Vitis vinifera*; Wu et al., 2013), and a 17.5 or 19.1% reduction in anthocyanins with a 8.4 or 6.8% reduction in sugars in cv. Sangiovese (Pastore et al., 2011, 2013). These results clearly indicate that the accumulation of sugars and anthocyanins are uncoupled under source limitation, and that carbon is preferentially allocated for sugar accumulation rather than anthocyanins. As mentioned in the introduction, several theories (CNB and GDB) have been developed in literature to describe the competition relationship between primary and secondary metabolites in plants (reviewed in Koricheva et al., 1998). Arnold et al. (2004) confirmed, with a series of elegant experiments, that the phenolic content and coloration of poplar (*Populus nigra* x *P. deltoides*) sink leaves is reduced by disrupted carbon flow from source to sink, namely reduced carbon availability. In cell suspensions of cv. Gamay Freaux that constructively produce anthocyanins, Guardiola et al. (1995) proved with a mathematical modeling approach that primary (sugars) and secondary (anthocyanins) metabolisms compete for carbon substrate when substrate is scarce. Our results provide a piece of evidence to the validity of CNB and GDB hypotheses in a productive sink (berries) and pave the way for modeling the sugars and anthocyanins accumulations in grape berry under various source-sink ratios. Interestingly, (Sadras and Moran, 2012) also reported the uncoupling between sugars and anthocyanins accumulation under high temperatures conditions. However, the biological mechanisms underlying this uncoupling effect should be different from those under carbon limitation, because temperature has direct effect on anthocyanin biosynthesis and degradation by modulating gene expression and enzyme activities of related enzymes (Mori et al., 2007). Few studies have been conducted to understand the inhibitory effect of source limitation on anthocyanin accumulation at protein and/or transcription levels. Two recent genome-wide transcriptome analyses showed that carbon limitation reduced the transcript abundance of UDP glucose:flavonoid-3-*O*-glucosyltransferase (UFGT) and GST4, which are known as important regulators of anthocyanin accumulation and transport (Pastore et al., 2011, 2013). A proteomic analysis showed that the abundances of chalcone synthase and dihydroflavonol reductase, which are both involved in anthocyanin pathway, were strongly reduced by source limitation (Wu et al., 2013). Our observed reduction in total anthocyanin in both cultivars under source limitation should also result from modifications in the key regulators of anthocyanin pathways, although further transcriptomic and proteomic experiments are needed to confirm these speculations.

Since it is known that different molecules of anthocyanins have different color hues and stabilities (He et al., 2010), we also studied the alteration of anthocyanin composition in response to carbon limitation. The concentration and composition of anthocyanins are different between Cabernet Sauvignon and Sangiovese berries under normal (carbon sufficient) condition; Cabernet Sauvignon berries had higher concentration of malvidin (tri-hydroxylated) derivatives and acylated anthocyanins, whereas Sangiovese berries were richer in cyanidin-3-glucoside (di-hyroxylated), and no acylated anthocyanins were found. These results are in agreement with a previous report (Mattivi et al., 2006). Carbon limitation increased the proportion of cyanidin-3-glucoside in Sangiovese. In the same cultivar, other authors (Filippetti et al., 2007; Pastore et al., 2011, 2013) also found that a decrease in source-sink ratio increased the proportion of cyanidin-3-glucoside. On the contrary, the proportion of the predominantly accumulated anthocyanin peonidin-3-glucoside (di-hydroxylated) in cv. Nebbiolo was decreased by low source-sink ratio (Guidoni et al., 2002). In Cabernet Sauvignon, we found the proportion of di-hydroxylated anthocyanins (cyanidin and peonidin derivatives) was reduced by carbon limitation. These results indicate that the modification in anthocyanin composition in response to source limitation is cultivar dependent. It is known that the ratio between di- and tri-hydroxylated anthocyanins is under the control of the relative activity of flavonoid 3^′^-hydroxylase (F3^′^H) and flavonoid 3^′^,5^′^-hydroxylase (F3^′^5^′^H) (Castellarin et al., 2006). Transcriptome analysis showed that carbon limitation increased the transcript abundance of F3^′^Hb, which is responsible for the biosynthesis of di-hydroxylated, and explained the observed modification in anthocyanin composition (Pastore et al., 2013). In addition to carbon source limitation, light exclusion can increase the ratio of di-/tri-hydroxylated anthocyanins (Guan et al., 2014); both water stress (Castellarin et al., 2007a,b) and high temperature decrease this ratio (Mori et al., 2007). Anthocyanin acylation is also known to be affected by light exposure and temperature (Tarara et al., 2008). In Cabernet Sauvignon, we also observed that source limitation significantly increased the proportion of acylated anthocyanins in compared to non-source limitation condition. The molecular regulation of anthocyanin acylation is largely unknown, although acylation can improve anthocyanin stability (Sarni et al., 1995; He et al., 2010). This differential responses between di- and tri-hydroxylated anthocyanins and between acylated and non-acylated anthocyanins can provide valuable potential implications in modulating wine quality. Further efforts are warranted to investigate why the activities or expression of F3^′^H and F3^′^5^′^H respond to source limitation differentially between cultivars and why acylated anthocyanins are preferably accumulated under source limitation.

Source and sink can communicate interactively and exert mutual influences on each other. When the source-to-sink ratio is reduced, the leaves (source) on the grapevine can increase leaf efficiency toward a compensation of their photosynthetic rate to meet the demand of berries (sink; Candolfi-Vasconcelos and Koblet, 1990; Petrie et al., 2003; Kliewer and Dokoozlian, 2005). We observed that the source limited vines increased their NCER per unit of LA compared to source sufficient vines. This was further confirmed by a higher content of chlorophyll (7% more in Sangiovese vines and 13.5% more in Cabernet Sauvignon vines), and a higher photosynthesis capacity measured on single leaf under optimal conditions. Similar effects of source limitation on leaf chlorophyll content have been observed by other authors (Candolfi-Vasconcelos and Koblet, 1990; Petrie et al., 2000b). Such photosynthetic compensation can explain why the source limited vines lost 69.6% of their LA although carbon fixation was reduced only 55.8% over berry ripening. However, it is clear that the compensation is partial, and this may be due to the fact that only the main leaves from the primary shoot were retained with all new growth being removed during our experiment. Mature leaves are less responsive to source-sink modulation (Candolfi-Vasconcelos et al., 1994b).

Comparing carbon fixation by leaves and carbon utilization by berries can provide a valuable estimation of the carbon balance between demand and supply. This information is essential to understand the physiology of vines and may help to develop mechanistic models (Poni et al., 2006b; Cola et al., 2014). However, this comparison is often missing in the source-sink modulation experiments due to the lack of suitable facilities to measure it. The whole-canopy gas exchange approach (Poni et al., 2014) makes it possible to monitor the seasonal NCER and to quantify the carbon fixed following the source-sink modulation. In addition, we followed the dynamics of sugar accumulation of berries and calculated the quantity of carbon used in the berries via a mathematic sugar accumulation function (Sadras et al., 2008). This provided a good estimate of carbon utilization in the most important sink (berries; Gutierrez et al., 1985; Coombe, 1989). The mathematical analysis of carbon-balance indicated that berry carbon utilization accounted for a higher proportion of fixed carbon for sugar accumulation under carbon limitation (73.4%) than under carbon adequacy (40.7%) during the sugar accumulation stages (54 to 81 DAF). This indicates that carbon allocation is not proportional to the carbon offer but with priorities to berries under source limitation, providing direct evidence to support the most applied assumption in grapevine carbon allocation models (Gutierrez et al., 1985; Bindi et al., 1996; Poni et al., 2006a; Pallas et al., 2008). The biological mechanisms behind this phenomenon have been poorly investigated. Pastore et al. (2011) showed that the transcript abundance of pyruvate decarboxylase isozyme 2 involved in glycolysis was reduced by a low source-sink ratio. However, we found that the carbon limitation increased enzyme activities involved in primary carbohydrate metabolisms (Dai et al. unpublished data). These increases in metabolic enzymes may confer higher sink strength and therefore allow berries to attract a higher proportion of carbon under source limitation. It is worth noting that our calculation do not consider the carbon utilization for maintenance and reserves, nor the potential contribution of reserve carbon remobilization (Gutierrez et al., 1985), but rather provides a quantitative indicator of carbon allocation. Therefore, the proportions close to 100% observed between 64 and 67 DAF in 3L vines are hardly realistic in real vines; instead they strongly indicate that carbon reserves are remobilized for berry sugar accumulation and/or vine maintenance. A similar reserve remobilization could explain the high carbon proportion that was observed when it was cloudy and the vine photosynthesis rate was extremely low. Although we did not quantify the reserve remobilization, Weaver (1963) reported that reserves (both soluble sugars and starch) in shoots were significantly reduced by source limitation in cv. Carignane and Zinfandel vines. Kliewer and Antcliff (1970) estimated that as much as 40% of the total sugars in berries may come from storage tissues of the vine. Using ^14^C-labeling, (Candolfi-Vasconcelos et al., 1994a) showed that carbon reserves from the woody storage tissues can be actively reallocated into berries under source limitations. Future use of ^14^C-labeling, whole-canopy net carbon exchange rate (NCER) measurement, and carbon content assessment in various tissues (leaf, shoot, wood, fruit, and root) would provide a valuable dataset for the quantification of the carbon balance and allocation analysis.

## Conclusion

Source limitation induced by leaf removal 1 week before veraison significantly reduced the concentration of sugars and anthocyanins but did not alter the concentration of organic acids in Cabernet Sauvignon and Sangiovese. Moreover, the magnitude of reduction was much greater in anthocyanins than sugars in response to source limitation, attesting to a decoupling between sugars and anthocyanins in both cultivars. Although the patterns of responses for sugars, organic acids, and total anthocyanins to source limitation are fairly consistent between cultivars, the modification of anthocyanin compositions is cultivar dependent. Therefore the grape berry manages the metabolic fate of carbon in such a way that sugar accumulation is maximally maintained at the expense of secondary metabolites (e.g., anthocyanins) under source limitation.

## Author Contributions

NB, SP, SD, EG, ZD designed and oversaw the research; NB, SP, ZD performed the research and analyzed data. NB, ZD drafted the manuscript; GH contributed to anthocyanin analysis; CR contributed to the analysis of primary metabolites; SP, SD, EG critically revised the manuscript. All authors read and approved the final manuscript.

## Conflict of Interest Statement

The authors declare that the research was conducted in the absence of any commercial or financial relationships that could be construed as a potential conflict of interest.
