# Supplementary material for: Differential responses of sugar, organic acids and anthocyanins to source-sink modulation in Cabernet Sauvignon and Sangiovese grapevines
Source: Front Plant Sci. 2015 May 29;6:382. doi: 10.3389/fpls.2015.00382 (PMC4448006; doi:10.3389/fpls.2015.00382)
Supplement: Supplementary file 1 [file Image_1.PDF]

frontiers

Supplementary Material

Differential responses of sugar, organic acids and anthocyanins to source-sink modulation in Cabernet Sauvignon and Sangiovese grapevines

Natalia Bobeica[1,2], Stefano Poni[2], Ghislaine Hilbert[1], Christel Renaud[1], Eric Gomès[3], Serge Delrot[3], Zhanwu Dai[1]*

[1] INRA, ISVV, EGFV, UMR 1287, Univ. Bordeaux, Villenave d'Ornon, France.
[2] Fruit Culture and Viticulture Section, Department of Sustainable Crop Production, Università Cattolica del Sacro Cuore, Piacenza, Italy.
[3] ISVV, INRA, EGFV, UMR 1287, Univ. Bordeaux, Villenave d'Ornon, France.

*** Correspondence:** Zhanwu Dai, INRA, ISVV, EGFV, UMR 1287, Univ. Bordeaux, 210 Chemin de Leysotte, F-33140 Villenave d'Ornon, France.
zhanwu.dai@bordeaux.inra.fr

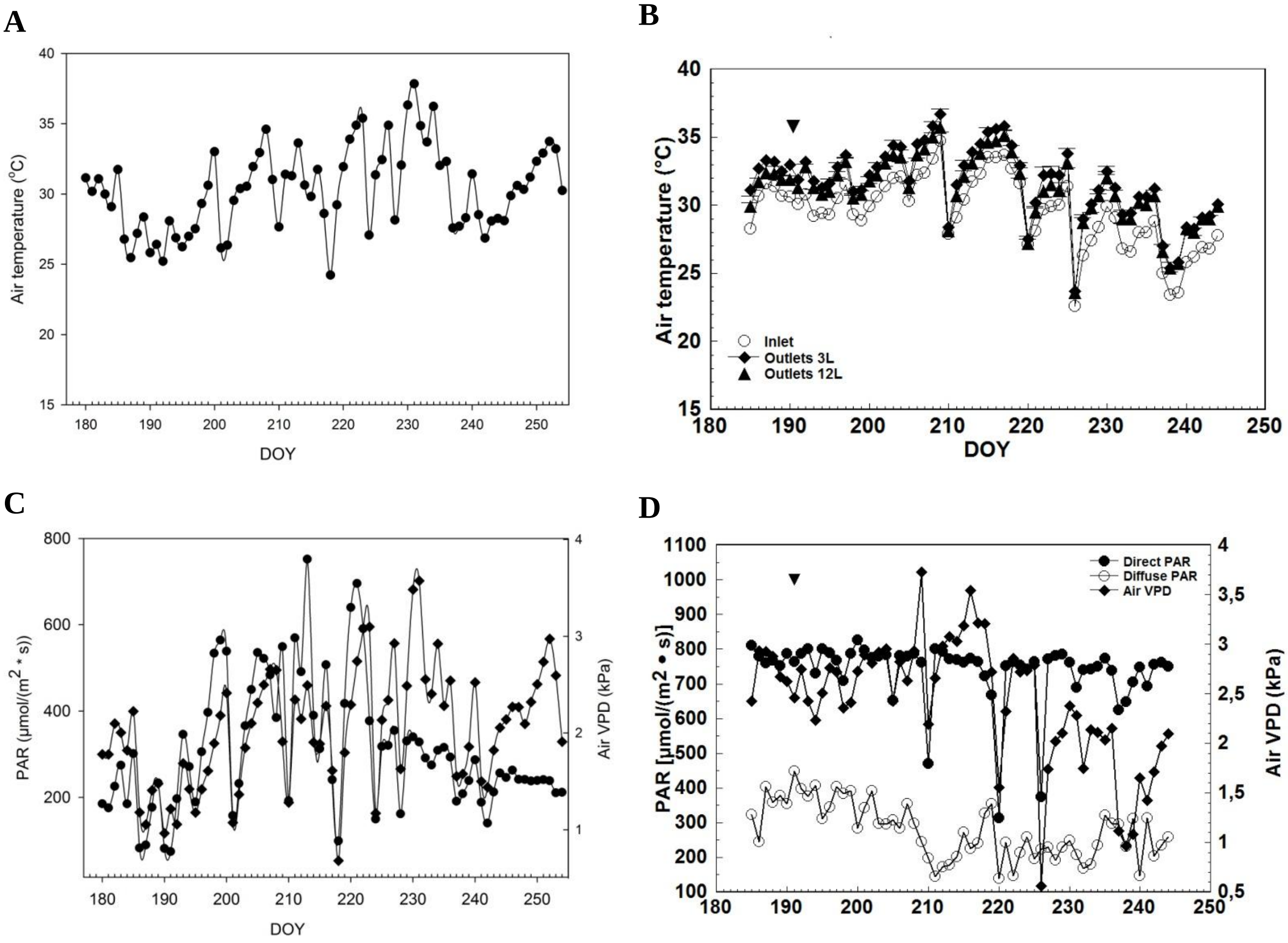

Supplementary Figure 1. Seasonal trends of (A) air temperature and (B) air vapour pressure deficit (VPD) and photosynthetically active radiation (PAR) measured in a semi-control greenhouse where Cabernet Sauvignon fruit cuttings were grown; (C) inlet and outlet chamber air temperature, (D) air vapour pressure deficit (VPD), direct and diffuse photosynthetically active radiation (PAR) measured in the whole-canopy gas exchange system where Sangiovese grapevines were grown. In (C) and (D), the solid triangle represent date of treatment.
